# Larval green and white sturgeon swimming performance in relation to water-diversion flows

**DOI:** 10.1093/conphys/cou031

**Published:** 2014-08-23

**Authors:** Christine E. Verhille, Jamilynn B. Poletto, Dennis E. Cocherell, Bethany DeCourten, Sarah Baird, Joseph J. Cech, Nann A. Fangue

**Affiliations:** 1 Department of Wildlife, Fish, & Conservation Biology, University of California Davis, One Shields Avenue, Davis, CA 95616, USA

**Keywords:** Sturgeon, swimming, water diversion

## Abstract

Little is known of the swimming capacities of larval sturgeons, despite global population declines in many species due in part to fragmentation of their spawning and rearing habitats by man-made water-diversion structures. Larval green (*Acipenser medirostris*) and white sturgeon (*Acipenser transmontanus*) inhabit the highly altered Sacramento–San Joaquin watershed, making them logical species to examine vulnerability to entrainment by altered water flows. The risk of larval sturgeon entrainment is influenced by the ontogeny of swimming capacity and dispersal timing and their interactions with water-diversion structure operations. Therefore, the aim of this study was to describe and compare the ontogeny and allometry of larval green and white sturgeon swimming capacities until completion of metamorphosis into juveniles. Despite the faster growth rates and eventual larger size of larval white sturgeon, green sturgeon critical swimming velocities remained consistently, though modestly, greater than those of white sturgeon throughout the larval life stage. Although behavioural interactions with water-diversion structures are also important considerations, regarding swimming capacity, Sacramento–San Joaquin sturgeons are most vulnerable to entrainment in February–May, when white sturgeon early larvae are in the middle Sacramento River, and April–May, when green sturgeon early larvae are in the upper river. Green sturgeon migrating downstream to the estuary and bays in October–November are also susceptible to entrainment due to their movements combined with seasonal declines in their swimming capacity. An additional inter-species comparison of the allometric relationship between critical swimming velocities and total length with several sturgeon species found throughout the world suggests a similar ontogeny of swimming capacity with growth. Therefore, although dispersal and behaviour differ among river systems and sturgeon species, similar recommendations are applicable for managers seeking to balance water demands with restoration and conservation of sturgeons worldwide.

## Abstract (precis)

Larval sturgeon swimming capacity has never been assessed. We measured critical swimming velocity of larval green and white sturgeon, and summarized published juvenile critical swimming velocity data for all sturgeon species. Recommendations for anthropogenic water diversion facility flow management were developed from the data, emphasizing Californian green and white sturgeon conservation.

## Introduction

Several sturgeon life-history traits, such as longevity, late maturation, spawning migrations and long breeding intervals, make sturgeon species worldwide vulnerable to anthropogenic pressures (Rochard *et al.*, 1990; Billard and Lecointre, 2001; Gessner *et al.*, 2007; Mussen *et al.*, 2014). Many sturgeon species spend all or part of their lives in coastal and inland systems, where habitat fragmentation by dams and other water-diversion structures is pervasive (Rochard *et al.*, 1990; Billard and Lecointre, 2001; Williot *et al.*, 2002). These structures can impede safe passage of migratory and resident fishes, interrupt watershed connectivity and adversely affect populations of many fish species (Xenopoulos *et al.*, 2005; Arnekleiv *et al.*, 2007; Caudill *et al.*, 2007; Liermann *et al.*, 2012), including sturgeons (Liermann *et al.*, 2012). The ability of fish to navigate or avoid water-diversion structures is related to their behavioural responses to water flow (Hinch and Bratty, 2000) and swimming capacities (Hoover *et al.*, 2005; Swanson *et al.*, 2005; Adams *et al.*, 2007; Boysen and Hoover, 2009). Sturgeons, in comparison to other anadromous fishes, may be particularly susceptible to altered flows around water-diversion structures due to their reduced swimming capacities (Peake *et al.*, 1997; Deslauriers and Kieffer, 2012a) and unique behavioural responses to flow (Webb, 1986; Peake *et al.,* 1997; Parsons *et al.,* 2003; Hoover *et al.,* 2005; Deslauriers and Kieffer, 2012a; Mussen *et al.*, 2014). Due to the pervasiveness of water diversion from sturgeon rivers worldwide and the protected status of all 27 sturgeon species by at least one international or national government body (IUCN, 2014; SARA, 2014; USFWS, 2014), assessments of swimming abilities of sturgeons that encounter anthropogenic water-diversion structures are important to understanding potential impacts on sturgeon populations.

Green (*Acipenser medirostris*) and white sturgeon (*Acipenser transmontanus*) are anadromous and semi-anadromous fishes, respectively, that are protected in North America. Green sturgeon spawn in Oregon and California and are composed of at least two genetically distinct populations (Israel *et al.*, 2009b). The Northern Distinct Population Segment (DPS), which is classified as a species of concern by the National Oceanic and Atmospheric Administration (NOAA) of the USA, includes all populations that spawn in rivers north of the Eel River of northwest California (Adams *et al.*, 2007). Confirmed spawning locations for the Northern DPS are the Rogue River in Oregon and the Klamath River in northern California, though additional spawning locations are suspected (Adams *et al.*, 2007). The Southern DPS is classified as threatened under the Endangered Species Act (ESA), and all suspected and confirmed spawning locations are within the Sacramento–San Joaquin (S-SJ) watershed in California (Adams *et al.*, 2007). A review of the current distribution of green sturgeon and the spawning locations of both the Northern and Southern DPS has been provided by Beamesderfer *et al.* (2007).

White sturgeon are found in three major North American drainages, i.e. Fraser, Columbia and S-SJ. Populations in the Kootenay, Upper Fraser, Nechako and Columbia rivers are protected by the Canadian Species at Risk Act (SARA, 2014). The American ESA recognizes the Kootenay population as endangered, and though the S-SJ population is not classified, the American Fisheries Society identifies them as a conservation concern (Musick *et al.*, 2000). White sturgeon are less marine oriented, with more life-history variation compared with green sturgeon. For example, the Kootenay population is landlocked and appears to disperse downstream more slowly than all other white sturgeon populations, which are semi-anadromous (McCabe and Tracy, 1993; Kynard and Parker, 2005; Kynard *et al.*, 2010; McAdam, 2011). Details on the populations and distribution of white sturgeon throughout their range have been provided by Schreier *et al.* (2013).

Green and white sturgeon inhabiting the S-SJ watershed face a profoundly altered habitat. The hydrological regimen of the S-SJ watershed has been severely disrupted since the late 1800s, when hydraulic mining operations were pervasive throughout the central Sierra Nevada region (Cloern and Jassby, 2012). These changes have led to fish extinctions, extirpations (Moyle, 2002) and population declines (Stevens *et al.*, 1985; Kimmerer *et al.*, 2001; Moyle, 2002; Sommer *et al.*, 2011). Despite the imminent threats to native species, conservation actions in the S-SJ watershed are challenging due to heavy societal water demands and use of the watershed as a resource. The S-SJ watershed supplies water to 25 million people and 1 million hectares of farmland (Service, 2007), facilitated by the construction of over 3300 water-diversion structures (Herren and Kawasaki, 2001), which divert more than 40% of the watershed drainage from the river system (Cloern and Jassby, 2012). Although many of these structures are not monitored for fish entrainment (i.e. being drawn in), anadromous fishes must pass by these diversion structures as they migrate, and the larval and juvenile fish of several species may be most susceptible to entrainment into diversions (Danley *et al.*, 2002; Grimaldo *et al.*, 2009). The spawning and rearing habitats of green and white sturgeon are located in the S-SJ watershed, and both species are susceptible to entrainment by the water diversion pumps operating in this watershed (Adams *et al.*, 2007; Israel *et al.*, 2009a; Mussen *et al.*, 2014). Indeed, Californian water diversions have been implicated in the population declines of species such as Chinook salmon (*Oncorhynchus tshawytscha*; Moyle, 2002), delta smelt (*Hypomesus transpacificus*; Bennett, 2005), striped bass (*Morone saxatilis*; Stevens *et al.*, 1985) and green sturgeon (Mussen *et al.*, 2014). Additionally, entrainments of both green and white sturgeon are reported at state and federal pumping facilities (NOAA, 2005; Israel and Klimley, 2008; Israel *et al.*, 2009a), with up to 10 000 white sturgeon reported in some years (Israel *et al.*, 2009a).

For many fish species, relative year-class strength is most highly influenced by embryonic to larval stages (Bradford, 1992). Recruitment failure during these early life stages has been identified as a major bottleneck to other North American acipenserid species (Hardy and Litvak, 2004), and specifically to white sturgeon (Duke *et al.*, 1999; Hildebrand *et al.*, 1999). Larvae and juvenile green sturgeon appear to disperse downstream rapidly following emergence, after which they spend 0.5–4 years foraging throughout the watershed (Adams *et al.*, 2007). Between ∼0.5 and 1.5 years of age, seawater tolerance (Allen and Cech, 2007, 2009, 2011) and a preference for high-salinity water (Poletto *et al.*, 2013) develops, suggesting a predisposition to migrate to marine waters at this age. Laboratory studies of white sturgeon suggest that salinities of 20 ppt are stressful to juveniles (McEnroe and Cech, 1985; Tashjian *et al.*, 2007). On the Columbia River, juvenile white sturgeon migrate seasonally up- and downstream, but have not been observed further downstream than the associated estuary (Parsley *et al.*, 2008). Their intolerance of high salinities and migratory behaviour suggest that white sturgeon spend their entire juvenile life in their natal rivers. Therefore, anthropogenic alterations to their natal river systems, particularly for the dispersal and foraging stages of both larval and juvenile green and white sturgeon, are likely to have severe consequences at the population level. Little is known about the susceptibility of these two species to encounters with water-diversion structures during their larval life stage, and almost nothing is known of their ability to resist the altered water flows around diversions if they do encounter these facilities, though juvenile green sturgeon [30 cm and 150–200 days post-hatch (dph)] appear to be more vulnerable to impingement on diversion screens than white sturgeon of a similar size and age in sweeping flows of 20 and 37 cm s^−1^ (Poletto *et al.* 2014).

Given that the ability of fishes to navigate or avoid water-diversion structures is related to their swimming capacity (Hoover *et al.*, 2005; Swanson *et al.*, 2005; Adams *et al.*, 2007; Boysen and Hoover, 2009), one method to assess the susceptibility of fishes to altered water flows, such as those at or near water diversions, is to quantify critical swimming velocities (i.e. an index of prolonged swimming capacity, *U*_crit_; Brett, 1964; Beamish, 1978). To date, the prolonged swimming capacities of larval green and white sturgeon have never been assessed. However, studies suggest that both species forage most actively from ∼30 dph onwards and spend the majority of their time up to 30 dph inactively hiding within rocky substrate (Kynard and Parker, 2005; Kynard *et al.*, 2005). The increased movement during this active foraging stage is most probably accompanied by increases in both swimming capacity and the potential to encounter water-diversion structures. Therefore, assessments of swimming abilities of these species during life stages that encounter water diversions are important to the management of these devices to minimize their impacts on sturgeon populations. In light of the paucity of information regarding swimming capacities of larval white and green sturgeon and their susceptibility to entrainment into water-diversion structures, the aim of the present study was to describe and compare the ontogeny of green and white sturgeon prolonged swimming capacities until completion of metamorphosis into juveniles, as well as to describe the allometry of swimming capacity throughout the juvenile life phase using swimming data in the published literature. We hypothesized that the similar lifestyles during the larval stage of these two species would be reflected in comparable swimming capacities throughout the larval life stage, but that the larger egg size of green sturgeon (Deng *et al.*, 2002) would result in larger larvae and faster growth throughout the larval stage. Furthermore, the allometry of green and white sturgeon swimming performance was compared with those of other sturgeon species to investigate inter-species differences in the ontogeny of swimming capacity. Finally, understanding the ontogeny of swimming capacity in green and white sturgeon of early dispersal and migration age can be used to inform conservation managers of appropriate protective water-diversion flow limitations specific to the S-SJ watershed and according to season and location.

## Materials and methods

Green and white sturgeon were reared in the laboratory at the Center for Aquatic Biology and Aquaculture, University of California, Davis (UC Davis). White sturgeon, obtained from Sterling Caviar and spawned in April 2012 and May 2013, were offspring of the F3 descendants of wild-caught Sacramento River sturgeon. Green sturgeon were tank spawned from first-generation domestic Klamath River broodstock in November 2011 and April 2013 following the methodology outlined by Van Eenennaam *et al.* (2001, 2012). For both species, the broodstock were spawned at 15°C, and eggs were reared at this temperature until 1 dph, when they were acclimated to 18 ± 0.5°C well water. At the onset of exogenous feeding (∼10–12 dph), green and white sturgeon larvae were fed *ad libitum* with Rangen (Buhl, ID, USA) salmonid starter moist feed. For each spawn, larvae were split into two 1-m-diameter flow-through tanks supplied with 18 ± 0.5°C, aerated well water (dissolved oxygen >8.2 mg l^−1^) creating flows of <5 cm s^−1^ and reared under a natural photoperiod (Van Eenennaam *et al.*, 2001, 2012). Fish handling, rearing and swimming tests were performed in agreement with the UC Davis Institutional Animal Care and Use Committee protocol no. 17017.

### Swimming performance

Protocols assessing *U*_crit_ were carried out at 18.5 ± 0.5°C. Due to their small size and frequent feeding requirements (Mohseni *et al.*, 2006), larval fish were fasted for 3–4 h before an individual fish was randomly chosen from alternating tanks and introduced into the swimming flume for a 30 min acclimation period. Swimming flumes were either a 1.5 l cylindrical flume or a 5 l rectangular, flat-bottomed flume (Loligo^®^ Systems, Tjele, Denmark) equipped with a motor and a variable frequency driver. During the acclimation period, water velocity was 1 cm s^−1^. After 30 min, water velocity was increased to 10 cm s^−1^ and then increased in increments of 5 cm s^−1^ every 5 or 10 min, depending on the spawn year. During each increment, fish were monitored for swimming behaviour. If fish became impinged, which was defined as contact between the downstream screen of the chamber and a third of their body for any duration or less than a third of their body for 30 s, timers and flow were stopped for 2 min. After the 2 min rest, timers were restarted, and flow was resumed at the velocity at the time of impingement. On the third impingement at the same velocity increment, or failure to recommence swimming after the 2 min break, the fish was considered fatigued (Allen *et al.*, 2006a). If a fish failed to successfully swim continuously through the first swimming velocity step by either holding station (avoiding swimming in the current) or impingement, that fish was considered to have low behavioural motivation to swim. These fish were not included in *U*_crit_ calculations, but are reported in the results as non-participants.

Preliminary tests with sturgeon larvae of different sizes and ages dictated that the time interval and swimming flume size and design be modified to achieve successful swimming throughout larval development. The 2013 spawn (green and white sturgeon measured at ages 20–42 dph) were swum in the 1.5 l cylindrical swimming flume for 5 min intervals. The 2011 green sturgeon and 2012 white sturgeon spawns (measured at ages 34–60 dph) were swum in the 5 l rectangular, flat-bottomed flume (Hoover *et al.*, 2011) for 10 min intervals. Comparisons of *U*_crit_ for 30–40 dph 2013 spawns (swum in the 1.5 l tunnel at 5 min intervals) with the same-aged 2011 or 2012 spawns (swum in the 5 l tunnel at 10 min intervals) using a one-way ANOVA did not differ in either species (*F*_1,22_ = 1.053, *P* = 0.316 for green sturgeon; and *F*_1,34_ = 0.541, *P* = 0.467 for white sturgeon).

Manufacturer calibration of the 1.5 l swimming flume was verified using the dye technique recommended by Loligo^®^. For each dial setting, red food colouring was injected via the effluent flush line using a 60 ml syringe, and the time for the dye front to travel 20.5 cm to the downstream flow straightener was measured using a stopwatch accurate to the hundredth of a second. The 5 l swimming flume was calibrated using a portable Marsh-McBirney water flowmeter (model 201D; Hach, Loveland, CO, USA), set to a 2 s time constant. For each dial setting on the flume motor, three separate measurements of flow were recorded, and the mean for each dial setting was calculated. Critical swimming velocity was calculated according to the formula:

$$U_{\mathrm{crit}}=V_{f}+V_{i}(T_{f}/T_{i}),$$

where *V*_f_ is the highest velocity at which the fish swam for the entire 10 or 5 min interval; *V*_i_ is the velocity increment (5 cm s^−1^); *T*_f_ is the duration of time the fish swam at the highest velocity attempted; and *T*_i_ is the time increment (10 or 5 min; Brett, 1964). Absolute *U*_crit_ was expressed in centimetres per second, and relative *U*_crit_ was calculated by dividing absolute *U*_crit_ of individual fish by total length (TL; in centimetres) of that fish, and expressed as body lengths per second (BL  s^−1^). The small fish size relative to tunnel cross-sectional area (≤5%) precluded the requirement for solid blocking effect adjustments (Bell and Terhune, 1970). Values of *U*_crit_ for the 72 green and 87 white sturgeon reported here were used both in the regression of larval *U*_crit_ values (in centimetres per second) with days post-hatch and in determination of the allometric exponents described in the ‘*Data analysis and statistics*’ section below.

### Growth

Following completion of swimming tests, all fish were euthanized by buffered anaesthetic overdose (MS-222, 1 g l^−1^) and the wet mass (in grams), girth (circumference at the opercula, in centimetres) and TL measured.

### Data analyses and statistics

Wet mass, TL and *U*_crit_ of larval green and white sturgeon, 20–60 dph, were evaluated for species differences by comparing the linear regression of TL, mass and absolute *U*_crit_ (in centimetres per second) with days post-hatch and *U*_crit_ (in centimetres per second) with TL.

In order to explore the allometric relationship between TL and relative *U*_crit_ (in body lengths per second) and absolute *U*_crit_ (in centimetres per second) in green and white sturgeon, we independently combined the larval green and white sturgeon *U*_crit_ values measured in this study with other *U*_crit_ data (N. A. Fangue, unpublished data) and published data mined from the literature for white and green sturgeon of larger sizes (Table 1). Important *U*_crit_ study parameters, such as temperature, velocity and time increments and fish age, are also summarized. The relationship between relative *U*_crit_ (in body lengths per second) and TL was fitted to a power function, and the allometric exponents for green and white sturgeon were independently determined as the slope of the linear regression of the log of TL vs. the log of *U*_crit_ (in body lengths per second). Although not included in calculations of allometric exponents, published *U*_crit_ data for seven other sturgeon species from nine studies were plotted for comparison with green and white sturgeon data (Table 1).

**Table 1:** Summary of values used for green and white sturgeon allometric exponent determinantions and literature values of *U*_crit_ for other sturgeon species

Species	TL [cm (mean ± SEM)]	*U*_crit_		*n*	Temp. (°C)	Speed increment (cm s^−1^)	Time interval (min)	Age (dph)
		BL s^−1^	cm s ^−1^					
Siberian sturgeon	58.4 (0.6)^1^	1.8 (<0.1)	105.5 (0.0)	4	24	10	10	600
	64.3 (0.9)^1^	1.7 (0.1)	106.3 (0.1)	7	24	10	10	600
Shortnose sturgeon	7.1 (<0.1)^2^	3.2 (0.2)	22.3 (0.6)	71	15	3	20	y-o-y
	19.4 (0.1)^2,3^	1.5 (0.1)	29.5 (1.3)	6	10–25	5	30	255
Lake sturgeon	55^4^	1.2	65		14		10	
Amur sturgeon	18.8 (0.3)^5^	2.0 (0.1)	36.8 (1.9)	18	20	0.25 BL s^−1^	30	
Chinese sturgeon	13.7 (2.0)^6^	2.6 (<0.1)	36.0 (5.0)	2	16–25	10	20	75–195
	24.5 (2.4)^6^	2.3 (0.1)	55.5 (2.5)	2	10–25	10	20	75–360
	35.3^6^	2.0	70.0	1	10–16	10	20	255–360
	40.5^6^	2.1	85.0	1	10–16	10	20	255–360
	54.8 (1.3)^1^	1.4 (<0.1)	77.8 (1.5)	7	24	10	10	600
	62.2 (0.7)^1^	1.3 (<0.1)	81.8 (3.8)	5	24	10	10	600
Pallid sturgeon	21.4 (0.3)^7^	1.7 (0.1)	35.9 (1.2)	8	20	5	30	180
Shovelnose sturgeon	23.1 (0.3)^7^	1.6 (1.2)	37.0 (1.4)	2	20	5	30	180
	57.0 (0)^8^	1.79 (0.2)	102.0 (14.0)	2	16	10	15	
	67.2 (1.4)^8^	1.4 (0.2)	90.9 (14.8)	3	16	10	15	
Green sturgeon	4.3 (0.2)^9a^	8.5 (0.4)	35.7 (1.7)	32	18–19	5	5	20–42
	6.5 (0.2)^9b^	7.1 (0.2)	45.3 (1.5)	40	18–19	5	10	34–60
	15.4 (0.6)^10a^	2.9 (0.1)	43.2 (1.3)	25	18–19	10	20	73–177
	22.1 (0.4)^10a^	2.2 (0.1)	48.1 (1.3)	27	18–19	10	20	73–177
	22.2 (0.6)^11^	2.4 (0.1)	52.9 (1.2)	20	18–19	5	5	
	34.7 (0.6)^10b^	1.4 (0.1)	4.8 (1.5)	22	18–19	10	20	73–177
	44.1 (0.7)^10b^	1.0 (0.1)	44.9 (4.0)	9	18–19	10	20	73–177
	49.4 (0.6)^12^	1.2 (0.5)	57.5 (2.5)	53	18–19	10	30	320–360
	68.3 (2.7)^13^	1.2 (0.1)	79.2 (4.9)	11	19	10	20	340–360
White sturgeon	4.7 (0.2)^9a^	5.5 (0.2)	25.2 (1.1)	43	18–19	5	5	20–42
	8.0 (0.4)^9c^	4.6 (0.2)	35.3 (1.4)	44	18–19	5	10	34–60
	24.8 (0.8)^11^	2.6 (0.1)	64.2 (1.6)	23	18–19	5	5	
	34.2^14^	1.6	56.4	1	11–12.5	5	15	
	38.3 (0.3)^11^	1.8 (0.1)	69.2 (2.2)	3	18–19	5	5	

Abbreviation: BL, body length; dph, days post-hatch; TL, total length; *U*_crit_, critical swimming velocity; y-o-y, young of the year.

^1^Qu *et al.* (2013). ^2^Deslauriers and Kieffer (2012a). ^3^Deslauriers and Kieffer (2011).

^4^Peake *et al.* (1995), cited by Adams *et al.* (1997). ^5^Cai *et al.* (2013). ^6^He *et al.* (2013).

^7^Adams *et al.* (2003). ^8^Adams *et al.* (1997). ^9a^Present study, 2013 spawned.

^9b^Present study, 2011 spawned. ^9c^Present study, 2012 spawned.

^10a^Allen *et al.* (2006a) saltwater-intolerant size range.

^10b^Allen *et al.* (2006a) saltwater-tolerant range.

^11^N. A. Fangue, unpublished data. ^12^Miller *et al.* (2014).

^13^Mayfield and Cech Jr (2004). ^14^Counihan and Frost (2011).

Linear regressions were performed using lm in R (http://www.r-project.org; see Table 2 for regression table), and statistical significance was considered at α = 0.05 for all analyses.

**Table 2:** Regression table for relationships between total length (TL; in centimetres), mass (in grams) and critical swimming velocity [*U*_crit_ (in body lengths per second)] and days post-hatch (dph) of green and white sturgeon

	Variable	β (±SEM)	*t*	*P* value
TL	dph	0.189 (0.010)	12.99	<0.001
	species	−2.503 (0.530)	−4.72	<0.001
	species × dph	0.091 (0.013)	6.85	<0.001
*F*_3,159_ = 311; adjusted *r*^2^ = 0.852; *P* < 0.001				
Mass	dph	0.130 (0.009)	5.62	<0.001
	species	−3.576 (0.565)	−6.33	<0.001
	species × dph	0.113 (0.0142)	8.00	<0.001
*F*_3,159_ = 146.2; adjusted *r*^2^ = 0.729; *P* < 0.001				
*U*_crit_	dph	0.673 (0.068)	8.40	<0.001
	species	−7.977 (4.426)	−1.80	0.073
	species × dph	−0.086 (0.111)	−0.77	0.441
*F*_3,159_ = 82; adjusted *r*^2^ = 0.598; *P* < 0.001				
*U*_crit_	TL	2.686 (0.339)	9.45	<0.001
	species	−4.125 (3.456)	−1.19	0.234
	species × dph	−1.836 (0.573)	−3.20	0.002
*F*_3,159_ = 103.9; adjusted *r*^2^ = 0.598; *P* < 0.001				

A multiplication sign signifies an interaction.

## Results

### Growth

**Figure 1.**
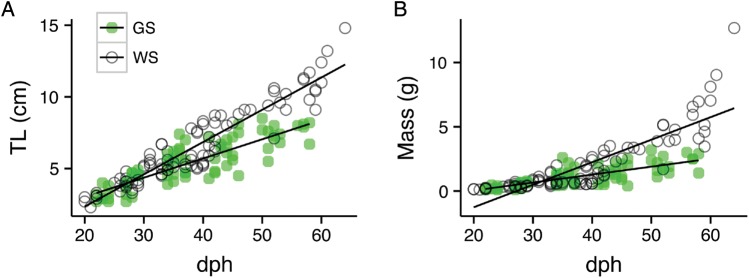
Ontogeny of length (TL, in centimetres; **A**) and mass (in grams; **B**) in larval green (GS, green circles, *n* = 72) and white sturgeon (WS, open circles, *n* = 87) from 20 to 60 days post-hatch (dph). The equations for the total length vs. days post-hatch regressions were *y* = 0.35 + 0.134*x* with an *r*^2^ of 0.715 and *y* = −2.16 + 0.225*x* with an *r*^2^ of 0.884 for larval green and white sturgeon, respectively. The equations for mass vs. days post-hatch regressions were *y* = −1.19 + 0.062*x* with an *r*^2^ of 0.586 and *y* = −4.77 + 0.175*x* with an *r*^2^ of 0.732 for larval green and white sturgeon, respectively.

Although the green and white sturgeon were a similar size early in the larval stage, the growth rate was greater for larval white than green sturgeon, resulting in larger white sturgeon at 25 and 32 dph for TL and mass comparisons, respectively (Fig. 1A and B). The equations for the total length vs. dph regressions were *y* = 0.35 + 0.134*x* and *y* = −2.16 + 0.225*x* for larval green and white sturgeon, respectively. The equations for mass vs. days post-hatch regressions were *y* = −1.19 + 0.062*x* and *y* = −4.77 + 0.175*x* for larval green and white sturgeon, respectively (Fig. 1A and B). Overall, white sturgeon rates of increase in mass and TL with age were similar, whereas for green sturgeon the TL increased at double the rate of mass (Fig. 1A and B).

### Swimming performance

Larval green and white sturgeon appeared to possess similar motivation to swim in the swim tunnel. Success rates for achieving continuous swimming in the swim tunnel for at least one interval were 87 and 86%, respectively, for green sturgeon (2011 spawned) and white sturgeon (2012 spawned), and 79 and 78%, respectively, for green and white sturgeon spawned in 2013.

**Figure 2.**
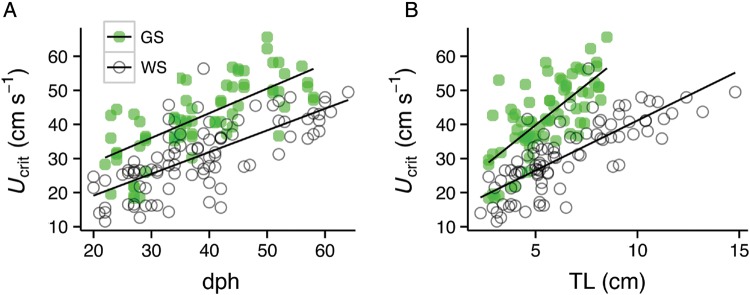
Ontogeny of larval green and white sturgeon critical swimming velocity (*U*_crit_, in centimetres per second) vs. days post-hatch (**A**) and total length (**B**) through the larval life stage. The equations of the regressions for *U*_crit_ (in centimetres per second) vs. days post-hatch were *y* = 14.34 + 0.724*x* with an *r*^2^ of 0.469 and *y* = 6.36 + 0.638*x* with an *r*^2^ of 0.497 for larval green (*n* = 72) and white sturgeon (*n* = 87), respectively. The equations of the regressions for *U*_crit_ (in centimetres per second) vs. days post-hatch were *y* = 15.97 + 4.766*x* with an *r*^2^ of 0.508 and *y* = 11.84 + 2.930*x* with an *r*^2^ of 0.603 for larval green (*n* = 72) and white sturgeon (*n* = 87), respectively.

Despite the slower growth of larval green sturgeon, ontogeny of absolute *U*_crit_ (in centimetres per second) did not differ between larval green and white sturgeon (Fig. 2A). The slope of increase in *U*_crit_ (in centimetres per second) with days post-hatch did not differ significantly between green and white sturgeon (*P* = 0.073; Table 2), but, due to a significantly larger intercept (*P* < 0.001), green sturgeon *U*_crit_ (in centimetres per second) was consistently greater than that of white sturgeon at the same age (Fig. 2A). The slope of increase in *U*_crit_ (in centimetres per second) with TL also did not differ significantly between green and white sturgeon (*P* = 0.234; Table 2), but there was a significant interaction between species and TL (*P* = 0.002; Table 2). Similar to the relationship between *U*_crit_ (in centimetres per second) and days post-hatch, the intercept of the relationship between *U*_crit_ (in centimetres per second) and TL for green sturgeon was also significantly greater than that for white sturgeon (*P* < 0.001; Table 2), resulting in green sturgeon *U*_crit_ (in centimetres per second) being consistently greater than that of white sturgeon at the same TL (Fig. 2B).

### Green and white sturgeon *U*_crit_ allometry

**Figure 3.**
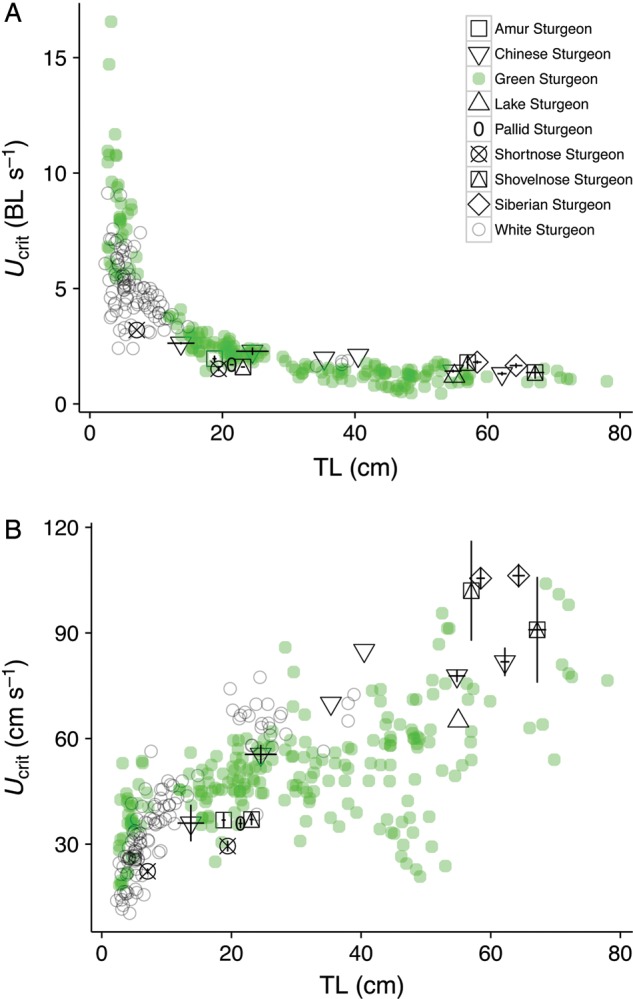
Allometry of green and white sturgeon and other *Acipenser* and *Scaphirhynchus* species' (see Table 1 for citations) relative critical swimming velocity [*U*_cri__t_, in body lengths (BL) per second; A] and absolute *U*_crit_ (in centimetres per second; **B**) vs. total length using data from this experiment and published and unpublished sturgeon *U*_crit_ data. The *U*_crit_ points for other sturgeon species represent means (±SEM), and citations and values are listed in Table 1. The temperature range across all studies was 10–25°C. Green and white sturgeon relative *U*_crit_ (in body lengths per second) changed with TL according to the function *y* = 3.34*x*^−0.8^^2^ (*r*^2^ = 0.89; *F*_1,234_ = 1997; *P* < 0.001) and *y* = 2.31*x *^−0.4^^2^ (*r*^2^ = 0.47; *F*_1,93_ = 84.5; *P* < 0.001), respectively.

As predicted, relative *U*_crit_ (in body lengths per second) vs. TL in juvenile green and white sturgeon decreased with increasing TL, and the relationship took the form of a power function (Fig. 3A). Also as expected, absolute *U*_crit_ (in centimetres per second) increased with TL for both species (Fig. 3B). The allometric exponent, which is the slope of decrease in relative *U*_crit_ (in body lengths per second) with growth, was greater for green (−0.83) than white sturgeon (−0.42). In sturgeons <5 cm long, green sturgeon relative *U*_crit_ (in body lengths per second) was twice that of white sturgeon. However, due to the more rapid decrease in green sturgeon relative *U*_crit_ (in body lengths per second) with increasing length, white sturgeon relative *U*_crit_ (in body lengths per second) began to exceed that of green sturgeon in fish that were between 12 and 20 cm TL. When relative *U*_crit_ values (in body lengths per second) of other sturgeon species were plotted with the green and white sturgeon allometric curves, both larval green and larval white sturgeon <20 cm in length appeared to have greater *U*_crit_ (in body lengths per second) than those of other sturgeon species of the same size (Fig. 3A). This difference disappeared in sturgeons >20 cm in length.

## Discussion

This first assessement of the swimming capacities of larval green and white sturgeon revealed biological differences between the two species, which alone are not expected to dictate remarkably different conservation strategies around water-diversion structures. Furthermore, we report the first attempt at determining the allometric relationship between swimming capacity and size in sturgeons, in which we found potential inter-species differences.

Although there are no published swimming capacity values for larval green and white sturgeon with which to compare *U*_crit_ results, the sizes of green sturgeon reported here generally fell within the mass and length ranges reported in previous studies performed at similar temperatures. Green sturgeon at the end of the larval stage (60 dph) were of similar masses (range, 1.5–2.7 g) and lengths (range, 6–7.5 cm) to previously reported late-larval green sturgeon masses (range, 1.5–3 g) and lengths (range, 6.2–9.4 cm; Deng *et al.*, 2002; Allen *et al.*, 2006b). White sturgeon, in contrast, tended to be slightly larger than previously reported. Although the early larval (20 dph) white sturgeon weight range of 0.2–0.4 g overlapped with white sturgeon of a similar age (20–30 dph) of 0.09–0.23 g (Deng *et al.*, 2003, 2009), late larval white sturgeon were slightly larger than previously reported. White sturgeon metamorphosing into juveniles (45 dph) in a previous study averaged 4.5 cm in length and had a mean weight of 1 g (Deng *et al.*, 2002), compared with the length and weight ranges of 7–9 cm and 2.5–3.2 g, respectively, for white sturgeon of a similar age in the present study. The larger size of late larval stage white sturgeon in this study is consistent with previously reported inter-family and inter-individual variability for green and white sturgeon (Van Eenennaam *et al.*, 2005; Linares-Casenave *et al.*, 2013) which may explain the ontological differences between the two species observed here. Replication of larval ontogeny studies is needed to strengthen support for these differences.

All larval growth studies discussed here, including this one, were performed at water temperatures ranging from 18 to 19°C. The effects of temperature on the growth of larval green and white sturgeon between 20 and 60 dph have never been assessed, but the growth of older, larger green sturgeon (>144 dph) increases with increasing temperature up to 15°C (Mayfield and Cech, 2004). We expect that similar effects of temperature on growth would occur in larval sturgeons. Therefore, given that size influences *U*_crit_ (Figs 2 and 3), temperature would most probably affect *U*_crit_ of larval sturgeons. This effect would probably also be influenced by the direct effects of temperature on swimming performance, which result in increased swimming speeds at higher temperatures, up to a high-temperature limit (Mayfield and Cech, 2004; Allen *et al.*, 2006a; Deslauriers and Kieffer, 2012b).

Absolute swimming capacity (i.e. expressed in centimetres per second) generally increases with increasing body size in fishes (Bainbridge, 1960; Brett, 1965; He, 1986), and absolute *U*_crit_ measured in both larval green and larval white sturgeon increased with size, as expected (Figs 2B and 3B). Also as expected, absolute *U*_crit_ values of larval green and larval white sturgeon increased at similar rates with growth through the larval life stage. However, *U*_crit_ values of larval green sturgeon aged 20–60 dph were consistently ∼10 cm s^−1^ greater than those for white sturgeon of the same age (Fig. 2A).

Unlike absolute swimming capacity, relative swimming capacity (i.e. expressed as body lengths per second) tends to decrease with size according to a power relationship in fishes (Bainbridge, 1960; Brett, 1965; He, 1986). The exponent of the power function relating maximal sprint swimming speed with body length has been determined as −1.09, −0.58 and −0.71 for sprinting common dace (*Leuciscus leuciscus*), rainbow trout (*Oncorynchus mykiss*) and goldfish (*Carassius auratus*), respectively (Bainbridge, 1960). For prolonged swimming capacity, the allometric exponent appears to range from −0.43 to −0.53, based on measurements on sockeye salmon (*Oncorynchus nerka*; calculated from Brett, 1965) and saithe (*Pollachius virens*; He, 1986). To our knowledge, the relationship between swimming capacity and body size has not been examined for any sturgeon species. Considering the unique body and tail morphology of these basal actinopterygians, which theoretically results in inefficient swimming kinetics (Webb, 1986; Qu *et al.*, 2013), we hypothesized that sturgeon species would possess a unique allometric exponent.

The body and tail shape of sturgeons differs from that of the typical, faster swimming actinopterygians and more closely resembles that of chondrichthyans. Differing from the more derived actinopterygian homocercal caudal fin, the sturgeon caudal fin is heterocercal, which, for lake sturgeon, has been shown to generate 66% less thrust than for the homocercal caudal fin of trout (Webb, 1986). Further reductions in acipenserid swimming efficiency arise from increased drag due to the sturgeon spindle-shaped body form and rough body surface (Webb, 1986), in comparison to the smooth surfaces and streamlined fusiform body shape of more derived fishes. This inefficiency in body design results in reduced burst, prolonged and sustained swimming capacities compared with those of salmonids (Peake *et al.*, 1997; Deslauliers and Kieffer, 2012a)*.* In combination with the behavioural tendency of sturgeons to station hold at high water velocities (Webb, 1986; Parsons *et al.,* 2003; Hoover *et al.,* 2005; Deslauliers and Kieffer, 2012a), these aspects of sturgeon morphology have the potential to alter the allometric exponent for the length–swimming capacity relationship for sturgeons compared with other actinopterygians.

Indeed, the allometric exponent for green, but not white sturgeon, as determined by relating *U*_crit_ and length of larval sturgeons from this study and juvenile sturgeons from previous studies, differed from that of other actinopterygians. The allometric exponent for all known *U*_crit_ (in body lengths per second) data for larval to 80-cm-long green and white sturgeon shows the allometric relationship between *U*_crit_ (in body lengths per second) and length of white sturgeon to have an exponent of −0.42, which is more like that of sockeye salmon (calculated from Brett, 1965) and saithe (He, 1986), compared with the exponent of −0.83 for green sturgeon, which more closely resembles the exponent for sprint swimming in common dace and goldfish (Bainbridge, 1960). Although these dissimilarities could be an artifact of the lack of *U*_crit_ values for 40- to 80-cm-long white sturgeon, they may also reflect differences in the ontogeny of green and white sturgeon prolonged swimming capacities.

Sometime between the completion of metamorphosis into juveniles (∼60 dph and at a TL of 7–8 and 9–11 cm for green and white sturgeon, respectively) and the onset of green sturgeon tolerance of full-strength sea water (∼130 dph and at a TL of ∼25 cm; Allen and Cech, 2007; Allen *et al.*, 2009, 2011), white sturgeon *U*_crit_ (in centimetres per second) began to exceed that of green sturgeon. This transition appears to be due to a shallower slope of increase in *U*_crit_ with length of green compared with white sturgeon during the early juvenile stage. The timing of this transition is consistent with previously reported evidence of a seasonal reduction in swimming capacity of green sturgeon undergoing physiological changes associated with preparation for downstream migration to estuarine or marine waters (Allen *et al.*, 2006a). More swimming data on white sturgeon of the 40–80 cm size range are required to determine whether this difference between green and white sturgeon *U*_crit_ is maintained throughout the life of these two species.

Although the paucity of published *U*_crit_ values for other sturgeon species across a significant size range prevents comparison of green and white sturgeon allometric exponents with those of other *Acipenser* species, early juvenile green and white sturgeon appear to have superior swimming capacities compared with other *Acipenser* species at this life stage. The *U*_crit_ (in centimetres per second) of Chinese sturgeon (*Acipenser sinensis*), pallid sturgeon (*Scaphirhynchus albus*), shovelnose sturgeon (*Scaphirhynchus platorynchus*), Amur sturgeon (*Acipenser schrenckii*) and shortnose sturgeon (*Acipenser brevirostrum*) with lengths of ∼20 cm or less lie below the green and white sturgeon allometric curves (Table 1 and Fig. 3A and B). Sturgeons >30 cm long appear to lie on the green sturgeon allometric curve. Thus, despite their potentially higher prolonged swimming capacity during the larval and early juvenile stage, green and white sturgeon have similar swimming capacities to other *Acipenser* species during later juvenile and adult life stages. It should be noted, however, that comparisons of *U*_crit_ values among studies have inherent challenges, because test temperatures (Mayfield and Cech, 2004; Allen *et al.*, 2006a) and end-point criteria for exhaustion, as well as the chosen time intervals and speed increments of the tests (Beamish, 1978; Hammer, 1995), are not standardized and can have substantial effects on *U*_crit_ measurements. It is possible that methodological differences may affect exhaustion end-points, thereby influencing *U*_crit_ values across *Acipenser* studies.

The stronger swimming capacities of larval and early juvenile green and white sturgeon may reflect demands for relatively vigorous swimming activity during early life stages of these species. Laboratory-based studies suggest that early juvenile white sturgeon from the Sacramento River undergo an active dispersal, with strong swimming behaviour (Kynard and Parker, 2005). Late larval green sturgeon have also been shown to swim actively up- and downstream while foraging in laboratory-based studies, and early juveniles actively migrate downstream to over-wintering grounds (Kynard *et al.*, 2005). Chinese sturgeon, in contrast, discontinue laboratory-observed migratory behaviour early in their life history, before the larval life stage begins (Zhuang *et al.*, 2002), and pallid and shovelnose sturgeon both exhibit passive drifting migratory behaviour in the laboratory (Kynard *et al.,* 2002), which suggests that their foraging phase may not require a high swimming capacity. Shortnose sturgeon are amphidromous (Bemis and Kynard, 1997); this primarily freshwater lifestyle may not require the swimming capacity of the anadromous green and semi-anadromous white sturgeon. Contrary to this interpretation of anadromous sturgeons having greater swimming capacity than non-anadromous sturgeons during the early juvenile phase, Amur sturgeon, which are anadromous (Bemis and Kynard, 1997), possess a lower swimming capacity than green sturgeon at the early juvenile life stage. In the laboratory, this species exhibits migratory behaviour lasting much of their larval stage (Zhuang *et al.*, 2003). Perhaps Amur sturgeon also experience a drop in swimming ability related to physiological preparation for entry into saline waters that is seen in green sturgeon with the onset of migration to brackish and salt water (Allen *et al.*, 2006a). Thus, it seems that the strong swimming ability of larval and early juvenile green and white sturgeon could be reflective of their life-history strategies.

Based on the results of this study and previously published laboratory behavioural and field observational studies, we have developed seasonal recommendations of water flow velocities likely to overwhelm larval green and white sturgeon at water-diversion facilities. We caution that these recommendations are based solely on capacity to maintain position in water flows, and encourage them to be considered in conjunction with species- and life-stage-specific behavioural responses to water flows and water-diversion structures. Unfortunately, sturgeon water-diversion-structure behavioural interaction studies are limited; however, juvenile green sturgeon appear to lack avoidance behaviour when encountering water-diversion structures (Mussen *et al.*, 2014).

### Green sturgeon larval and juvenile migration and behaviour

The threatened Southern Distinct Population Segment of green sturgeon spawn primarily in the upper reaches of the Sacramento River from April to May (Adams *et al.*, 2007; Heublein *et al.*, 2008)*.* Based on laboratory studies, it appears that hatched green sturgeon, unlike other acipenserids, which are typically transiently pelagic immediately after hatch, exclusively hide in rocks at the river bottom until initiation of exogenous feeding at 10 dph. At this stage, they begin a 10 day nocturnally active dispersal downstream to foraging sites in the middle reaches of the river (Kynard *et al.,* 2005). Once dispersed to foraging sites, larval green sturgeon appear to forage nocturnally on the river bottom and hide in the rocks during the day until ∼100 dph or the autumn, when they begin another active downstream migration (Kynard *et al.,* 2005). At this age, green sturgeon have demonstrated tolerance of and preference for near full-strength seawater (Allen and Cech, 2007; Allen *et al.*, 2009, 2011; Poletto *et al.,* 2013) and are therefore likely to migrate to the S-SJ Delta or San Pablo and San Francisco bays. The physiological preparation process for this migration from fresh to saltwater has been associated with reductions in *U*_crit_ of juvenile (0.5- to 1.5-year-old) green sturgeon (Allen *et al.,* 2006a).

### Green sturgeon conservation recommendations

During their initial dispersal migration in spring, the tiny 10 dph larval green sturgeon are probably vulnerable to being overwhelmed by water-diversion intake flows. At 20–25 dph (∼2 cm TL), green sturgeon *U*_crit_ ranged from 20 to 53 cm s^−1^, and the linear regression predicted a mean *U*_crit_ of 29 cm s^−1^. Swimming capacity consistently increased with days post-hatch (Fig. 2), which suggests that younger, migrating green sturgeon are unlikely to have swimming capacities this high. Therefore, nighttime flows at water-diversion structures likely to be encountered by green sturgeon in the upper and middle reaches of the Sacramento River from May through the summer should be limited to 29 cm s^−1^, assuming that they detect the diversion flows and can avoid them behaviourally. Mussen *et al.* (2014) showed that larger juvenile green sturgeon are easily entrained in a simulated water-diversion intake structure.

By 55 dph, when green sturgeon were ∼7 cm long and expected to remain nocturnally foraging in the middle reaches of the river, mean *U*_crit_ predicted from the linear regression increased 1.9-fold to 54 cm s^−1^ (Fig. 2) and ranged from 46 to 58 cm s^−1^. This suggests that diversion structures in the middle reaches of the Sacramento River should be limited to maximal velocities of 54 cm s^−1^ during the night from July until the following May spawn, assuming that they detect the diversion flows and can avoid them behaviourally.

The *U*_crit_ of juvenile green sturgeon changes slowly with growth, such that with a 6-fold increase in total length from 8 to 50 cm, mean *U*_crit_ increased only 1.2-fold, from 50 to 60 cm s^−1^ (Fig. 3B). In addition to this modest increase, *U*_crit_ values of 50-cm-long sturgeon are highly variable, ranging from 20 to 75 cm s^−1^, with nearly a quarter of the sturgeon at this size swimming < 40 cm s^−1^. As this size corresponds to the range exhibiting reductions in swimming capacity thought to be related to downstream migration to saltwater (Allen *et al.*, 2006a), a corresponding reduction in maximal diversion velocities to 40 cm s^−1^ may be important to protect migrating juvenile green sturgeon through the middle and lower reaches of the Sacramento River and the Delta and Bays in October and November (Mussen *et al.*, 2014).

Swimming capacity of green sturgeon larger than 70 cm has not been assessed. Therefore, lower Sacramento River and Delta diversion structures from which juvenile green sturgeon are expected to encounter flows should be limited to maximal flows of 54 cm s^−1^, assuming that they detect the diversion flows and can avoid them behaviourally.

### White sturgeon larval and juvenile migration and behaviour

Most Sacramento River white sturgeon spawning appears to be limited to between Colusa (river km 231) and Verona, California (river km 160; Kolhorst, 1976). Laboratory studies suggest that immediately upon hatching, white sturgeon hatchlings disperse passively downstream for 10 days (Kynard and Parker, 2005). From 10 to 28 dph, white sturgeon forage with gradually increasing activity and progress from hiding within rocky substrate to complete use of open bottom, additionally spending some time at the surface or within the water column (Kynard and Parker, 2005). Active foraging appears to continue until 50 dph, when white sturgeon behaviour in the laboratory suggests a second downstream migration (Kynard and Parker, 2005). Larvae have been found as far downstream as Suisun Bay, but such far-downstream larval sightings occur in high-flow years when larvae have probably been overwhelmed by flows and flushed downstream (Stevens and Miller, 1970).

### White sturgeon conservation recommendations

Hatchling white sturgeon are likely to be highly susceptible to entrainment into water-diversion structures during the passive dispersal phase, because these fish have virtually no ability to resist diversion flows during this life stage. Middle to lower Sacramento River diversion structures in the vicinity of white sturgeon spawning sites should be highly regulated when adults are spawning (February–May).

Due to their high foraging activity, 30 dph larval white sturgeon, which are located throughout the lower reaches of the Sacramento River from March to June, are likely to be vulnerable to entrainment into water-diversion structures. At 35 dph (∼5.5 cm TL), white sturgeon *U*_crit_ values ranged from 22 to 45 cm s^−1^, and the linear regression equation predicted a *U*_crit_ of 29 cm s^−1^ (Fig. 2), suggesting that the majority of the population, if able to detect and avoid diversion flows, would not be vulnerable to water-diversion flow rates lower than 29 cm s^−1^. Later in the summer, as larval white sturgeon grow and metamorphose into juveniles (60 dph, ∼10 cm TL), their swimming capacity, and therefore their ability to escape diversion structure flows, increased slightly to 41–48 cm s^−1^, with the linear regression predicting a *U*_crit_ of 45 cm s^−1^ (Fig. 2). At this developmental stage, some sturgeon have also been found in the Bay (Stevens and Miller, 1970) and, therefore, potentially the Delta as well. Therefore, lower Sacramento River and Delta diversion structures potentially encountered by foraging larval white sturgeon from March to June should be limited to maximal flows of 29 cm s^−1^. These limits can probably be increased further in the autumn to a maximum of 50 cm s^−1^ as fish grow and increase swimming capacity (Figs 2 and 3) until the next spawn in the following February. Although *U*_crit_ values of 115 cm s^−1^ were achieved by 95-cm-long (7 kg) white sturgeon (personal communication from Nguyen, Jackson, and Peterson), fish of this size are likely to be >1 year old. Through the winter months, neither size nor swimming capacity is expected to increase, so it is unlikely that white sturgeon spending their first winter in the Sacramento River are able to hold position in water flows >50 cm s^−1^.

We developed season-specific recommendations for flow limitations around water-diversion structures of the Sacramento River and its Delta by integrating laboratory-based findings on the ontogeny of green and white sturgeon with swimming capacity and behavioural information (Table 3).

**Table 3:** Overview of flow-tolerance limitations of green and white sturgeon throughout the Sacramento–San Joaquin watershed according to location and time of year, based on critical swimming velocity data

	Upper river	Middle river	Lower river/delta/bays
January	<50 cm s^−1^		
February		WS early larvae	
March			
April	GS early larvae		
May			
June	GS and WS 29 cm s^−1^		
July		WS 45 cm s^−1^	
August		GS 50 cm s^−1^	
September			
October	<50 cm s^−1^	GS 40 cm s^−1^	
November			
December			

Abbreviations: GS, green sturgeon; WS, white sturgeon. Green sections demarcate tolerable water velocities of ≥50 cm s^−1^; red sections demarcate presence of life stages which are predicted to be intolerant of even very low water velocities; and yellow sections signify recommended water flow velocity limitations to protect present life stages. Behavioural (e.g. avoidance) considerations are not part of this analysis, and they remain an important topic for future research.

Throughout the entire Sacramento River system from December to February, sturgeons present are unlikely to be overwhelmed by flows below 50 cm s^−1^. This period can be extended to April for the upper reaches, where green sturgeon tend to spawn. From February to June, populations of highly susceptible larval sturgeons with limited to no swimming ability are present within the river system. This is of particular concern in the middle to lower reaches from February until April, when young white sturgeon are expected to be drifting passively downstream from spawning sites. In the upper reaches, susceptible larval green sturgeon with limited swimming capabilities are expected to be present in April and May. By the end of May, green sturgeon larvae in the upper reaches are expected to have developed sufficient swimming ability to resist flows up to 29 cm s^−1^, but white sturgeon in the middle to lower reaches are not expected to develop similar swimming capacities until June. By July, although green sturgeon present throughout the upper to lower reaches are likely to be able to resist water flows >50 cm s^−1^, white sturgeon in the lower reaches remain limited to flows of 45 cm s^−1^ until September. In October, as juvenile green sturgeon develop saltwater tolerance and begin their downstream migration to the estuaries and bays, their swimming capacity drops, requiring a 2 month (October and November) 40 cm s^−1^ limit of flows around water-diversion structures in the middle and lower Sacramento River and the Delta and bays.

Similar conservation recommendations can be applied to other acipenserid species, with adjustments for dispersal behaviour, river site usage and behavioural responses to flows and diversion structures. The slightly greater absolute *U*_crit_ (in centimetres per second) of early juvenile green and white sturgeon compared with other sturgeon species suggests that acipenserids in general require lower velocities than green and white sturgeon around diversion structures, if they are to resist entrainment volitionally. However, it is also important to consider the behavioural responses of the different sturgeons and their life stages to water-diversion structures. The complete lack of published larval *U*_crit_ data for any acipenserid species before this study underscores the urgency for studies on this vulnerable life stage of this globally imperiled family.
